# Predictors of the effectiveness of an early medication change strategy in patients with major depressive disorder

**DOI:** 10.1186/s12888-019-2014-x

**Published:** 2019-01-14

**Authors:** Nadine Dreimüller, Stefanie Wagner, Alice Engel, Dieter F. Braus, Sibylle C. Roll, Stefan Elsner, André Tadić, Klaus Lieb

**Affiliations:** 1 Department of Psychiatry and Psychotherapy, University Medical Center of the Johannes Gutenberg-University Mainz, Untere Zahlbacher Straße 8, D-55131 Mainz, Germany; 2 Department for Psychiatry and Psychotherapy, HELIOS Dr. Horst Schmidt Kliniken, Wiesbaden, Germany; 3 Hospital for Psychiatry and Psychotherapy, Vitos Rheingau, Eltville, Germany; 4 Hospital for Psychiatry and Psychotherapy, Andernach, Germany; 5 Agaplesion Elisabethenstift, Department of Psychiatry, Psychosomatics and Psychotherapy, Darmstadt, Germany

**Keywords:** Major depressive disorder, Predictor, Antidepressant, Early improvement, Treatment outcome

## Abstract

**Background:**

Patients with Major Depressive Disorder (MDD) who are non-improvers after two weeks of antidepressant treatment have a high risk of treatment failure. Recently, we did not find differences in outcomes in non-improvers randomized to an early medication change (EMC) strategy compared to treatment as usual (TAU). This secondary analysis investigated possible predictors of higher remission rates in the EMC strategy.

**Methods:**

Of 192 non-improvers (i.e. decrease of ≤20% on the HAMD-17 depression scale) after a two-week treatment with escitalopram, *n* = 97 were randomized to EMC (immediate switch to high doses of venlafaxine XR) and *n* = 95 to TAU (continued escitalopram until day 28 with non-responders switched to venlafaxine XR). We first analyzed patient characteristics, psychopathological features and subtypes of MDD by logistic regression analyses as possible predictors of remission rates. In a second investigation, we analyzed the predictors, which showed a significant association in the first analysis before Bonferroni-Holm correction by chi-squared tests separated for treatment groups. All analyses were corrected by Bonferroni-Holm method.

**Results:**

The first analyses yielded no statistically significant results after correction for multiple testing. In the second analyses, however, patients with prior medication at study entry showed higher remission rates in EMC than in TAU (24.2% versus 8.6%, *p* = 0.017; Bonferroni-Holm corrected significance level: *p* = 0.025.). Furthermore, patients with a recurrent course of MDD benefited less from treatment as usual (*p* = 0.009; Bonferroni-Holm corrected significance level: *p* = 0.025). Age, sex, age of onset, psychiatric or somatic comorbidities, and other subtypes of MDD did not predict remission rates.

**Conclusions:**

Although in our first analysis we found statistically non-significant results, the second analysis showed significant differences in remission rates between patients with or without previous medication and in patients with recurrent MDD or the first depressive episode. It would therefore be valuable to examine in larger and prospective studies whether remission rates can be increased by quick escalation of treatment in certain subgroups of patients. Promising subgroups to be tested are patients who were previously medicated, and who show a recurrent course of MDD.

**Trial registration:**

clinicaltrials.gov Identifier: NCT00974155. Registered at the 10th of September 2009. Retrospectively registered.

## Background

Major Depressive Disorder (MDD) is a very severe, highly prevalent and very costly psychiatric disorder that is one of the leading causes of global burden of diseases because of its substantial morbidity and mortality [1–7]. Antidepressants are – apart from psychotherapy and psychosocial interventions – the main treatment option for MDD, especially for moderate and severe MDD [8]. However, about two thirds of patients do not benefit sufficiently from the first antidepressant. They require a switch to or an augmentation with a second substance [9]. Although antidepressants have been systematically administered for the treatment of MDD for more than 50 years, there is still uncertainty about the ideal time to assess the onset of antidepressants action. For decades, the common clinical view was that onset of antidepressant action appears with a delay of 2–4 weeks and can only be fully evaluated after 6–12 weeks [10–12]. Patients who do not show more than 20% improvement after two weeks of antidepressant treatment, however, have a high risk of later treatment failure ([13, 14], see also [15]). Based on this observation we performed a randomized controlled trial with 889 patients to determine whether an early medication change (EMC) strategy is superior to a guideline based treatment in MDD patients with non-improvement (defined as a reduction of ≤20% on the HAMD-17) after two weeks of antidepressant treatment. The results of the EMC study confirmed data from post-hoc analyses of clinical trials showing that early non-improvement identifies patients who may need alternate interventions. In the EMC group, most of the clinically relevant secondary outcomes like remission rate measured by the IDS (Inventory of Depressive Symptomatology [16]), time to remission, symptom response and absolute symptom decrease showed consistently advantageous results. Nevertheless, the results did not confirm the merits of the switch/adjunct therapy strategy, as only 24% of patients remitted according to the Hamilton Rating Scale for Depression (HAMD-17) [17], which was not significantly different form the remission rate in the TAU group [18].

With more than 227 possible symptom combinations, different subtypes and long-term courses, MDD is a very heterogeneous disorder. A previous meta-review of 754 studies in MDD-patients identified fifteen subtype models including symptom-based, etiologically-based, time of onset-based, gender-based and treatment response-based subtypes [19]. This heterogeneity may explain at least in part the disappointing treatment outcomes to antidepressants in patients with MDD and may possibly explain why the EMC strategy was not superior to TAU in our study. In fact, different subtypes of depression may need a subtype-specific treatment as previously suggested by our group and others [20–24]. However, only a few studies had tested specific treatment options for the identified subtypes yet [19, 25].

Based on the consideration that the heterogeneity of patients with MDD may explain why we did not find differences in outcomes between the EMC strategy and TAU in our earlier study [18], the aim of the current study was to investigate a limited set of MDD subtypes, comorbidities and patient characteristics as possible predictors of different remission rates in the EMC and the TAU group.

## Methods

### Participants and study procedure

Methods and design of the EMC trial have been described in detail previously [18, 26]. In short, the EMC trial was a phase IV, multi-center, multi-step, randomized, observer-blinded, actively controlled parallel-group clinical trial which was registered on the ClinicalTrial.gov database as NCT00974155, and is conducted following the Consolidated Standards of Reporting Trials guidelines (CONSORT); all study participants provided written informed consent at enrollment into the protocol treatment. The ethics committee at the State Chamber of Physicians of Rhineland Palatinate and the German Federal Institute for Drugs and Medical Devices (BfArM) approved the trial protocol. A Data and Safety Monitoring Committee (DSMC) supervised the trial progress to ensure safety of subjects and research integrity.

Eight hundred eighty-nine adult patients (age 18–65 and < 60 years at the time of the first depressive episode) – treated as inpatient or in a day hospital – at one of the eight participating centers in Germany with a primary diagnosis of nonpsychotic MDD (DSM-IV) [27] and a sum score ≥ 18 on the HAMD-17 at study entry were enrolled between September 2009 and March 2014. Minimal exclusion and broad inclusion criteria were used to maximize generalizability. Patients with a primary diagnosis of bipolar, psychotic, obsessive-compulsive, eating disorder or substance dependence (if it required inpatient detoxification) and female patients who were pregnant or breast-feeding were excluded from the study, as well as those with general medical conditions contraindicating the use of any protocol medication, or a clear history of non-response (≤50% reduction of HAMD-17) or intolerance in the current MDD episode to any protocol antidepressant. After inclusion and – if necessary – washout of not allowed drugs, 879 patients received the selective serotonin reuptake inhibitor (SSRI) escitalopram for 14 days (20 mg; mean dose = 19.1 ± 1.0 mg/day). Of those, 192 patients had no improvement, defined as a reduction of ≤20% on the HAMD-17 after 14 days of treatment. These non-improvers were randomly assigned to open treatment with the EMC strategy (*n* = 97) or treatment as usual (TAU; *n* = 95). In the EMC arm, treatment was immediately switched to venlafaxine XR for study days 15–56 with a starting dose of 75 mg and dose escalation in 75 mg steps every day depending on acceptability, (mean dose = 255.7 ± 62.9 mg/day). In case of sustained non-improvement on day 28, lithium augmentation (mean dose = 692.4 ± 84.7.3 mg) for days 29–56 was performed. In the TAU arm, escitalopram was continued for additional 14 days (mean dose = 19.1 ± 1.9 mg), and non-responders on day 28 were switched to venlafaxine XR (mean dose = 262.8 ± 62.6 mg) for four weeks, i.e. days 29–56.

### Possible predictors of treatment outcome

We assessed the following limited sets of predictors of treatment outcome:

i) patient characteristics (age, sex, age of onset, prior medication yes/no)
ii) recurrent course of MDD vs. first episode
iii) symptom-based subtypes of MDD: melancholic MDD, atypical MDD, anxious MDD, and MDD with suicidality
iv) comorbidity-based subtypes: comorbid axis I disorders, comorbid axis II disorders, relevant somatic comorbidities

The classification into MDD subtypes (melancholic and atypical) was done based on the IDS single items instead of the HAMD-17 items, since the IDS contains all the questions to diagnose a MDD episode as well as items relevant to melancholic or atypical symptom features by the DSM-IV [27]. The anxious subtype was classified based on the HAMD-17. Comorbid axis I disorders were assessed by the Mini-International Neuropsychiatric Interview (M.I.N.I.) [28], axis II disorders by the Structured Clinical Interview for DSM-IV Axis II Disorders (SCID-II) [29]. Somatic comorbidities were assessed by the Cumulative Illness Rating Scale (CIRS), which considers the severity of co-occurring medical conditions [30]. The CIRS addresses 12 organs or organ systems, the total score ranges from 0 to 56, based on a scoring from 0 to 4 as follows: 0, no problem; 1, minor current problem or significant history; 2, morbidity or moderate discomfort, requiring primary care treatment; 3, severe problem: constant significant discomfort, chronic problem difficult to control; 4, extremely severe problem, requiring immediate treatment: organ failure or severe functional impairment. In the CIRS sum score; we excluded the psychiatric subitem in order to separate psychiatric-somatic symptoms from somatic symptoms. Additionally, we assessed somatic symptoms of depression in the HAMD interview and the raters were trained only to assess somatic comorbidities in the CIRS, which are not a direct consequence of the current depression. We here present the impairment in the single CIRS subdomains and the severity level (range 0–4) of the item No. 11 of the HAMD-17 concerning “psychiatric somatic symptoms” (see Table 1).

**Table 1:** Clinical and sociodemographic characteristics of patients

Characteristic	EMC (*n* = 97)	TAU (*n* = 95)	*p-*value
*Patient characteristics*			
Age (mean +/− SD)	39.4 ± 11.5	38.9 ± 12.2	0.796
Female sex – *n* (%)	55 (57)	52 (55)	0.784
*Symptom-based subtypes - n* (%)			
Melancholic MDD	78 (80)	81 (85)	0.721
Atypical MDD	36 (37)	35 (37)	0.881
Anxious MDD	65 (67)	67 (71)	0.762
MDD with suicidality	80 (84)	75 (79)	0.389
1st episode - *n* (%)	30 (31)	31 (33)	0.924
*Severity of depression (IDS-C30)*			
Mean IDS-C30 score (mean +/− SD)	44.2 ± 8.7	45.3 ± 8.4	0.352
Mean IDS-SR30 score (mean +/− SD)	45.5 ± 10.3	45.5 ± 10.1	0.958
Axis I - *n* (%)	52 (54)	52 (55)	0.996
Axis II - *n* (%)	42 (43)	37 (39)	0.554
*Physical comorbidities CIRS*			
Total severity score ((mean +/− SD)	1.9 ± 2.1	1.7 ± 1.9	0.506
Cardiac	0.1 ± 0.4	0.1 ± 0.1	0.460
Hypertension	0.02 ± 0.1	0.02 ± 0.1	0.983
Vascular	0.2 ± 0.5	0.3 ± 0.6	0.136
Respiratory	0.4 ± 0.7	0.3 ± 0.6	0.339
ENT	0.1 ± 0.3	0.1 ± 0.5	0.402
Upper GI	0.1 ± 0.4	0.1 ± 0.1	0.470
Lower GI	0.1 ± 0.3	0.2 ± 0.5	0.195
Hepatic	0.01 ± 0.1	0.02 ± 0.2	0.645
Renal	0.1 ± 0.3	0.1 ± 0.4	0.543
Musculo-sceletal-integumentary	0.4 ± 0.9	0.3 ± 0.6	0.275
Neurological	2.1 ± 1.2	2.4 ± 1.4	0.334
Endocrine-metabolic	0.2 ± 0.5	0.2 ± 0.5	0.208
Psychiatric somatic symptoms (HAMD-17 item 11)	1.6 ± 1.0	1.8 ± 1.0	0.104

*SD* Standard deviation, *EMC* Early Medication Change, *TAU* Treatment as Usual, *MDD* Major Depressive Disorder, *IDS-30* 30-item Quick Inventory of Depressive Symptomatology, *C* Clinician–Rated, *SR* Self-Rating, *CIRS* Clinical incident rating scale, without the psychiatric somatic symptoms; Data concerning age, gender, 1st episode/recurrent MDD, severity of depression, psychiatric and physical comorbidities were published before in [18].

We used remission rates as endpoint in this study, defined as a HAMD-17 sum score < 7 points after 8 weeks. Blinded raters, who were unaware of the treatment group, administered the HAMD-17. They were extensively trained in the administration of the HAMD-17 before participation in the study [31].

### Statistical analyses

The analyses focused on the ITT population that comprised all randomized patients. For this analysis, dropouts before day 28 were counted as non-remitters; dropouts after day 28 were counted as remitters or non-remitters according to the last HAMD-17 sum score. We performed the analyses on an exploratory basis, since no literature was found concerning treatment response of subgroups of depressive patients in an EMC strategy and since this was a secondary analysis of the study with no power analysis and sample size estimation according to the addressed research question. First, we performed logistic regression analyses with remission as outcome and the treatment groups (EMC/TAU), the subtypes and the interaction (treatment group x subgroup) as criteria. We used the Bonferroni-Holm method to correct for multiple testing [32]. Instead of including all variables in one “family” for the “family wise error rate” (FWER), which would have meant to include 12 variables in the correction analysis, we separated them into the families/sets that were determined a-priori and listed under “Possible predictors of treatment outcome”. Therefore, we analyzed each of the four sets independent from each other, correcting for the number of comparisons performed within each of the four sets rather than the grand total number of comparisons. To further investigate the association between subtypes and treatment groups, we performed a second analysis. Here, Fisher’s exact tests at a two-sided significance level were used to compare remission rates between subtypes separated for treatment groups. In this analysis, we included all subtypes which showed a significant association in our first analysis before Bonferroni-Holm correction. These subgroup analyses were also corrected for multiple testing using the Bonferroni-Holm method. The three identified subtypes were part of different sets of predictors. All analyses were carried out using SPSS 23. Significance was set at *p* ≤ 0.05.

## Results

Possible predictors of remission were assessed in 192 patients classified as non-improvers on day 14 and randomized to either EMC (*n* = 97) or TAU (*n* = 95). As previously published [18], demographic and clinical characteristics of patients randomized to EMC or TAU were comparable at baseline. Dropouts were similar in both groups. Furthermore, both groups did not differ in MDD subtypes at baseline (Table 1). Patients suffered from mild to moderate physical comorbidities as assessed by the CIRS, in contrast to psychiatric somatic symptoms as assessed by item 11 (anxiety somatic (physiological concomitants of anxiety)) of the HAMD-17 which were much more present.

The logistic regression analyses with remission as outcome and the subtypes, the treatment groups (EMC, TAU) and their interaction (treatment group*predictor) as criteria, revealed no association between remission rates and patient characteristics (age, sex, age of onset).

**Fig. 1:**
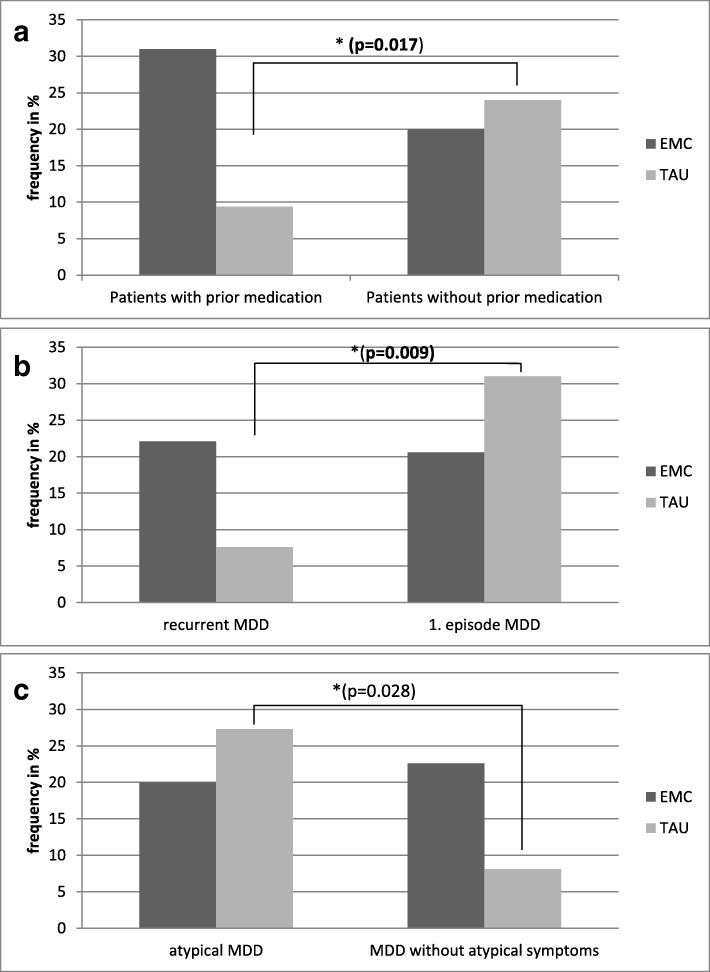
**a-c**: Remission rates at endpoint in patients randomized to EMC or TAU. a: In relation to prior medication. **a**: MDD: Major depressive disorder; EMC: Early Medication Change; TAU: Treatment as usual; * difference in remission Chi2-Test (df = 1) **bold** if significant. TAU arm: remission in patients with prior medication (8.6%), remission in patients without prior medication 24.3% ***p*** **= 0.017**. Bonferroni-Holm corrected significance level *p* = 0.025. In the EMC Arm: remission in patients without prior medication 17.1%, remission in patients with prior medication 31.2%; Chi^2^ = 0.656; df = 1; *p* = 0.456, Bonferroni-Holm corrected significance level: p = 0.025. **b**: In relation to a recurrent course of MDD or first episode. **b**: MDD: Major Depressive Disorder; EMC: Early Medication Change; TAU: Treatment as usual; * difference in remission Chi^2^-Test (df = 1) **bold** if significant. TAU-arm: remission in patients with recurrent MDD 7.6%, patients with first episode 31% ***p*** **= 0.009**; Bonferroni-Holm corrected significance level: p = 0.025. EMC-arm: remission in patients with recurrent MDD 22.1%, patients with first episode 20.6% *p* = 0.028; Bonferroni-Holm corrected significance level: p = 0.025; n.s. **c**: In relation to the presence of atypical MDD. **c**: MDD: Major Depressive Disorder; EMC: Early Medication Change; TAU: Treatment as Usual; * significant difference in remission Chi2-Test (df = 1) **bold** if significant. TAU-arm: remission in patients with patients with atypical MDD 27.8%, patients without atypical features 8.1% p = 0.028; Bonferroni-Holm corrected significance level: *p* = 0.025, n.s. EMC-arm: remission in patients with patients with atypical MDD 20.6%, patients without atypical features 22.6% *p* = 0.558; Bonferroni-Holm corrected significance level: *p* = 0.025; n.s

However, patients who were treated with another antidepressant directly before entering the study showed a not significantly different outcome in the EMC group (Odds ratio = 0.183; 95% confidence interval: 0.124–3.796; *p* = 0.031 without correction; Bonferroni-Holm corrected significance level *p* = 0.004, n.s., see Table 2). In the TAU arm patients without prior medication showed significantly higher remission rates than patients with prior medication (24.2% versus 8.6%; Chi^2^ = 7.958; df = 1; ***p*** **= 0.017**, Bonferroni-Holm corrected significance level: p = 0,025. See Fig. 1a). Regarding the course of MDD, patients with a recurrent MDD showed a not significantly higher remission rate in EMC than in TAU (Odds ratio = 0.061; 95% confidence interval: − 4.016 – − 0.229; *p* = 0.041; Bonferroni-Holm corrected significance level; *p* = 0.025; see Table 2). The second analysis confirmed that patients with their first depressive episode or a recurrent MDD were not significantly different in remission rates in the EMC group (Chi^2^ = 8.65; df = 1, *p* = 0.028; Bonferroni-Holm corrected significance level p = 0.025, n.s.), but in the TAU group. Here patients with the first depressive episode had significantly higher remission rates (31%) than patients with a recurrent MDD (7.5%) (Chi^2^ = 8.824; df = 1; ***p*** **= 0.009**; Bonferroni-Holm corrected significance level: p = 0.025; see also Fig. 1b).

**Table 2:** Association between possible predictors (separated for four sets of possible predictors as determined in the methods section), treatment groups (EMC/TAU) and remission at endpoint

Possible predictor	Criteria	Odds ratio (Expo [β])	*p*-value	Bonferroni holm corrected *p*-value^1^
Age	treatment group	0.398	0.413	0.007
	subtype	0.360	0.431	0.008
	subtype*group	1.013	0.673	0.017
Sex	treatment group	0.398	0.457	0.010
	subtype	0.760	0.807	0.050
	subtype*group	1.303	0.724	0.025
Age of onset	treatment group	0.228	0.159	0.005
	subtype	0.952	0.329	0.006
	subtype*group	1.033	0.318	0.006
Prior medication	treatment group	1.481	0.484	0.013
	subtype	7.600	0.087	0.005
	subtype*group	0.183	**0.031**	0.004
Course of depression (1. episode/recurrent MDD)	treatment group	0.325	**0.029**	0.017
	subtype	0.229	0.229	0.050
	subtype*group	0.061	**0.041**	0.025
Melancholic MDD	treatment group	0.962	0.956	0.050
	subtype	1.337	0.820	0.013
	subtype*group	0.539	0.460	0.006
Anxious depression	treatment group	0.700	0.532	0.007
	subtype	0.795	0.839	0.017
	subtype*group	0.785	0.748	0.010
Atypical MDD	treatment group	0.275	0.019	0.004
	subtype	0.174	0.174	0.005
	subtype*group	5.339	**0.032**	0.005
MDD with Suicidality	treatment group	0.933	0.922	0.025
	subtype	1.620	0.713	0.008
	subtype*group	0.518	0.430	0.006
Comorbid axis I disorder	treatment group	0.491	0.202	0.008
	subtype	0.712	0.761	0.025
	subtype*group	0.617	0.617	0.017
Comorbid axis II disorder	treatment group	0.561	0.195	0.007
	subtype	0.489	0.551	0.013
	subtype*group	1.084	0.921	0.050
CIRS sum score	treatment group	1.601	0.506	0.010
	subtype	1.352	0.175	0.006
	subtype*group	0.778	0.119	0.006

^1^ in B-H correction, results are defined as significant (**bold**) if the p-value of the regression analysis is lower or equal as the B-H corrected *p*-value; * logistic regression analyses; *MDD* Major depressive disorder, *CIRS* Cumulative Illness Rating Scale

Patients with a recurrent MDD did not significantly overlap with patients with prior medication. 43.1% of the patients with a first episode were taking antidepressants at study entry and 70.9% of patients with a recurrent MDD.

In the analyses of symptom-based subtypes, only in patients with an atypical form of MDD a relevant but not significant relationship between treatment group and subtype was found (Odds ratio = 5.339, 95% confidence interval: 0.068–3.597, *p* = 0.032; Bonferroni-Holm corrected *p* = 0.005; n.s. see Table 2). Patients with or without atypical symptoms did not significantly differ in remission rates in both groups (EMC: Chi^2^ = 0.809; df = 1; *p* = 0.558; Bonferroni-Holm corrected significance level *p* = 0.025; n.s); TAU: (27.3% versus 8.1%; Chi^2^ = 5.533; df = 1; *p* = 0.028; Bonferroni-Holm corrected significance level: p = 0.025; n.s. See also Fig. 1c). No differences between EMC and TAU were found in the other MDD subtypes, melancholic MDD, anxious MDD and MDD with suicidality.

Regarding the comorbidity-based subtypes, no significant differences between EMC and TAU were found, neither in comorbid axis I disorders, nor in comorbid axis II disorders or in relevant somatic comorbidities (see Table 2).

## Discussion

We investigated whether certain patient characteristics, psychopathological features or subtypes of MDD may serve as predictors for remission after an EMC treatment strategy in patients with MDD. The analyses were performed in patients who were non-improvers after two weeks of escitalopram treatment and who were treated by either an EMC strategy or TAU. Our first analyses yielded non-significant results after Bonferroni-Holm correction for multiple testing. Only for patients on prior medication, with recurrent MDD and without atypical features of MDD we found preliminary hints of a better treatment outcome in the EMC group. Therefore, we performed a second analysis with these promising markers. In these analyses we found evidence that patients who were treated with another antidepressant before study entry and who had a recurrent course of MDD may benefit more from an early optimization of medication instead of a treatment according to current guidelines. The other interesting finding that patients with an atypical MDD benefited more from TAU could not be confirmed after Bonferroni-Holm correction.

As significant results were only found in the second analyses, we interpret our study as negative. Although we report here a negative finding, we nevertheless believe that the results are interesting and relevant for clinicians because this is the first analysis of differential treatment outcomes in subgroups of non-improvers to escitalopram treated by a quick escalation of antidepressant therapy as compared to TAU.

In general, a comparison of our study results to earlier studies in which possible predictors of treatment outcome in MDD were investigated is difficult. Nearly all of these studies investigated patient characteristics at study entry as possible predictors of treatment outcome after 6–12 weeks of treatment independent of a non-improver status after two weeks of treatment. A more comparable analysis was performed in one of our recent publications of the EMC study sample, which showed that patients who are non-improvers to two weeks of escitalopram treatment are more likely to show non-remission, if they have melancholic features of MDD [24]. However, a melancholic subtype of MDD did not explain a different treatment outcome to EMC or TAU as analyzed in the present study, which only focused on the 192 non-improvers of our EMC study and not on the total sample of 889 patients [18].

Data on the clinical utility of MDD-subtypes in predicting antidepressant treatment response are inconsistent. Whereas one recent, uncontrolled and rather small study showed that a melancholic subtype of MDD predicted a better treatment response to fluoxetine [33], other studies did not find an effect of a melancholic subtype on treatment outcome [25, 34–36]. In particular, the so far largest trial, the iSPOT-D trial, which investigated treatment response to escitalopram, sertraline or venlafaxine in 1008 patients with different MDD subtypes, found no differences in treatment response according to MDD subtypes [25]. A mixed picture is also found in the literature for response in patients with atypical features of MDD [37]. Whereas some studies suggest that patients with atypical depression have lower remission rates than patients without atypical features [38], other studies found no differences [39] or found that lower remission rates were no longer significant after adjustment for pretreatment baseline differences [40]. Another preliminary finding of our study is that patients with recurrent MDD may have higher remission rates in the EMC arm as compared to TAU. A recent study of the CO-MED Study Team showed that early-onset chronic/recurrent MDD was associated with a more severe clinical picture, but did not seem to be useful for predicting differential treatment response to antidepressant medication [41]. Due to the small subgroup of patients with early onset of MDD in our sample of early non-improvers to escitalopram (25 patients), the statistical power was too low to replicate the finding of the CO-Med Study. The reason why patients with a recurrent course of MDD may possibly benefit more from the EMC treatment is unclear, and this possible association should be confirmed in future studies.

The preliminary finding that patients who were on prior antidepressant medication before inclusion into the study may benefit more from an EMC strategy supports recommendations of recent treatment guidelines [12], which do not recommend intraclass switches of antidepressants in case of non-remission. Since most patients with prior antidepressant medication (57.5%) [42] were on a SSRI before entering our EMC trial, continued treatment with escitalopram represents an intraclass switch (SSRI to SSRI). As a switch to a dual antidepressant may be more effective [43], the earlier switch to venlafaxine XR may explain why patients in the EMC arm showed higher remission rates than patients in the TAU arm which who switched later and only in case of a non-response to escitalopram.

Our finding that patient characteristics such as age and sex failed to show a significant impact on remission rates is in line with earlier studies [44]. Although it is well known that patients with comorbid psychiatric or somatic disorders have worse treatment outcomes as compared to patients without comorbidities [9, 45, 46], antidepressant treatment response was not different in the EMC and the TAU arm. Alternate strategies have, therefore, to be established and tested for those patient groups in order to enhance treatment outcomes.

### Clinical and research implications

Most studies so far have investigated whether certain subtypes of MDD or patients’ characteristics at treatment initiation lead to different treatment outcomes at study end in depressed patients. The innovative nature of our study, in contrast, was to examine possible predictors of treatment outcome in a group of non-improvers to two weeks of escitalopram treatment, randomized to different treatment strategies.

Although, the identified markers have to be validated in larger samples, our study may have clinical relevance despite the missing statistical significance. The clinical predictors may help clinicians to identify patients, who benefit from an early medication change and as a consequence reduce personal suffering and economic costs by shortening the duration of therapy, or for whom it may be advisable to stay longer on the chosen therapy. In clinical care, knowledge of outcome predictors may help forming realistic expectations of treatment outcome and considering alternative approaches for individuals who are less likely to benefit from routine first-line treatment. The presence of one or more of the identified predictors should alert clinicians to tailor their treatment approach in order to maximize the chances of remission. Since the current study was unique in its design and treatment algorithm, but not sufficiently powered for the research question addressed, well powered prospective studies examining moderators which alter response to different treatment strategies are needed to proof whether similar conclusions can be found with regard to the preliminary predictors found in this study. Further research inspired by our study may additionally investigate how markers of clinical course and patient characteristics can be combined with different treatment strategies to improve treatment outcome. Such studies may in the end help to increase the low remission rates in the treatment of MDD. We hope we can stimulate other researchers to carry out similar studies - if possible with larger sample sizes and thus higher statistical power. Summarizing data from several such studies may hopefully result in statistically significant findings.

### Limitations

An important limitation of this study is that it was a secondary analysis on an exploratory basis which was not powered to answer our research question. In fact, some subgroups of non-improvers were very small which limited the chance to detect small effects; e.g. the group of comorbid anxiety disorders or comorbid trauma history. Since also other studies might have been underpowered to detect significant predictors, comparisons to other study results have to be done very carefully. A further limitation comprises the open delivery of treatment and the lack of a placebo control. Raters for the assessment of the efficacy outcomes, however, were blinded to group assignment and protocol medication; additionally, a potential bias by the subjects’ expectations has been addressed by neutral oral and written patient information not favoring any of the treatment strategies. Without a placebo control, we cannot be sure that any of the treatment strategies was specifically effective and the results were due to the pharmacologic effects of the medication. However, the switch to placebo after an initial failed antidepressant treatment would have limited the acceptance and generalizability and would have raised ethical concerns.

## Conclusions

Although this study yielded non-significant results, we believe that our results are nevertheless of relevance for hypothesis building and planning of future treatment studies in patients with MDD. In particular, it would be valuable to further conduct prospective and sufficiently powered studies to examine whether remission rates can be increased by quick escalation of treatment in certain subgroups of patients. Promising subgroups to be tested are patients who were previously medicated, who show a recurrent course of MDD and who show no signs of atypical depression. The successful conduction of such studies may help to improve the currently often disappointing treatment situation of patients who are often treated for long periods of time with the same antidepressant [42] as those treatments are not stratified according to clinical predictors [47].

## Abbreviations

BfrarM: German Federal Institute for Drugs and Medical Devices
BMBF: German Federal Ministry of Education and Research
CIRS: Cumulative Illness Rating Scale
DSM-IV: Diagnostic and Statistical Manual on Mental Disorders
EMC: Early Medication Change
HAMD-17: Hamilton Rating Scale for Depression
IDS: Inventory of Depressive Symptomatology
ITT: intention to treat
MDD: Major depressive disorder
mg: milligram
n: number
n.s.: not significant
p: significance level
SSRI: selective serotonin reuptake inhibitor
TAU: Treatment as usual
